# From intersection names to street rankings in Kyoto: Comparing ranking methods for incomplete pairwise data

**DOI:** 10.1371/journal.pone.0358233

**Published:** 2026-09-17

**Authors:** Takashi Teramoto

**Affiliations:** Department of Data Science, Kyoto Women’s University, Kyoto, Japan; Zhejiang Agriculture and Forestry University: Zhejiang A and F University, CHINA

## Abstract

Rankings constructed from incomplete pairwise comparisons may depend on the method used, yet applied studies often report a single ordering without quantifying disagreement among methods. Kyoto’s intersection names provide both a methodological example and a subject of broad public interest. Central Kyoto has an approximately grid-like street network, and many intersections are named by combining the names of one east–west and one north–south street. The order of the two street names is not fixed. Inspired by a newspaper article asking which street is the “strongest,” we treat the street mentioned first in an intersection name as the winner and the second as the loser. When an intersection bears a neutral name containing neither street name, the two streets are assigned a draw. The dataset consists of 59 observed comparisons, 51 decisive outcomes and eight draws, among the 64 possible pairs formed by eight east–west and eight north–south streets. The remaining five street pairs, for which no intersection name exists, are treated as missing comparisons. Rankings were calculated using Colley, Keener, and Markov chain-based methods. Nishioji-dori ranked second under the Colley method and first under both the Keener and Markov methods. Simple averaging of the normalized scores placed Nishioji-dori first, closely followed by Kujo-dori. Although the methods broadly agreed on the top-ranked streets, their score spacing and lower positions differed. HodgeRank further localized residual disagreement among the methods. This study demonstrates ranking and rank aggregation for an incomplete bipartite comparison network while showing how familiar civic data can connect quantitative research with public interest in Kyoto’s history and everyday geography.

## Introduction

Rankings based on pairwise outcomes are simpler when every entity is compared with every other entity. Many real-world datasets, however, are incomplete or structurally constrained. Sports competitions, hyperlink networks, preference data, and other comparison systems may contain entities that never meet directly. Their ordering must therefore be inferred through chains of common opponents or neighbors. The Colley, Keener, and Markov chain-based methods address this problem from different perspectives [1–4]. Because they assign different weights to direct outcomes and opponent strength, they do not necessarily produce identical rankings. Previous comparative studies have shown that linear algebra-based ranking methods may differ in their sensitivity, stability, and the properties they satisfy [5,6].

Applied ranking studies nevertheless often report a single final order without examining whether it is stable across methods. Rank aggregation can summarize several outputs [1,7,8], but an overall ranking score alone does not indicate where or how the methods disagree. HodgeRank provides a least-squares framework for decomposing pairwise edge flows into a globally consistent gradient component and for analyzing the remaining inconsistency [9]. A particularly revealing setting is an incomplete bipartite comparison network, in which entities within the same class are never directly compared. The stability of rankings and the localization of disagreement among methods in such networks have received less attention in applied case studies.

Kyoto’s intersection-naming system supplies a naturally occurring example. Central Kyoto has an approximately grid-like street pattern. Intersections are commonly named by combining one east–west and one north–south street name, but the order is not fixed. For example, the east–west street “Shijo-dori” precedes the intersecting street in “Shijo-Karasuma,” whereas the north–south street “Karasuma-dori” precedes Gojo-dori in “Karasuma-Gojo.” Historical accounts relate this variation to neighborhood organization, traffic, and the standardization of tram-stop and intersection names [10–12]. A local newspaper article proposed treating the street named first as the winner of a metaphorical match and asked whether Shijo-dori was Kyoto’s “strongest” street [13].

This naming convention creates a methodological problem that extends beyond the local question. East–west streets are compared only with north–south streets, so streets running in the same direction never meet directly. Simple win totals cannot account for opponent strength, and the resulting order may depend on the ranking method. Previous discussions of Kyoto intersection names have been primarily historical or journalistic [10–13]. These articles have neither analyzed street or intersection naming conventions using multiple ranking methods nor distinguished the ordering common to those methods from their disagreement. The present study addresses this gap by formulating the name-order data as an incomplete bipartite comparison network.

The contribution is therefore not a new ranking method. It is a reproducible analytical framework that (i) converts a culturally embedded naming practice into pairwise comparison data, (ii) compares three established ranking methods, (iii) tests whether the leading group is robust to the aggregation scale, and (iv) quantifies the residual disagreement among methods.

This study has three objectives. First, it constructs pairwise comparison data from the ordering of street names at Kyoto intersections. Second, it compares the rankings obtained by the Colley, Keener, and Markov chain-based methods. Third, it obtains an overall ranking by simple averaging and examines disagreement among the three methods using HodgeRank. The scope is limited to eight east–west and eight north–south streets, with 59 observed comparisons among the 64 possible street pairs. The word “stronger” is used only as an operational label for name precedence and does not denote the intrinsic importance of a street such as traffic volume, economic importance, historical value, or cultural significance. The remainder of the paper is organized as follows. Materials and methods describes the historical context of Kyoto’s streets and intersection names, the construction of the comparison data, and the three ranking methods. Results presents the rankings obtained by each method, their simple average, and the HodgeRank results. Discussion interprets the agreements and differences among the methods in relation to the Kyoto case, and Conclusion summarizes the overall findings.

## Materials and methods

### Study context: Kyoto’s streets and intersection names

**Fig 1:**
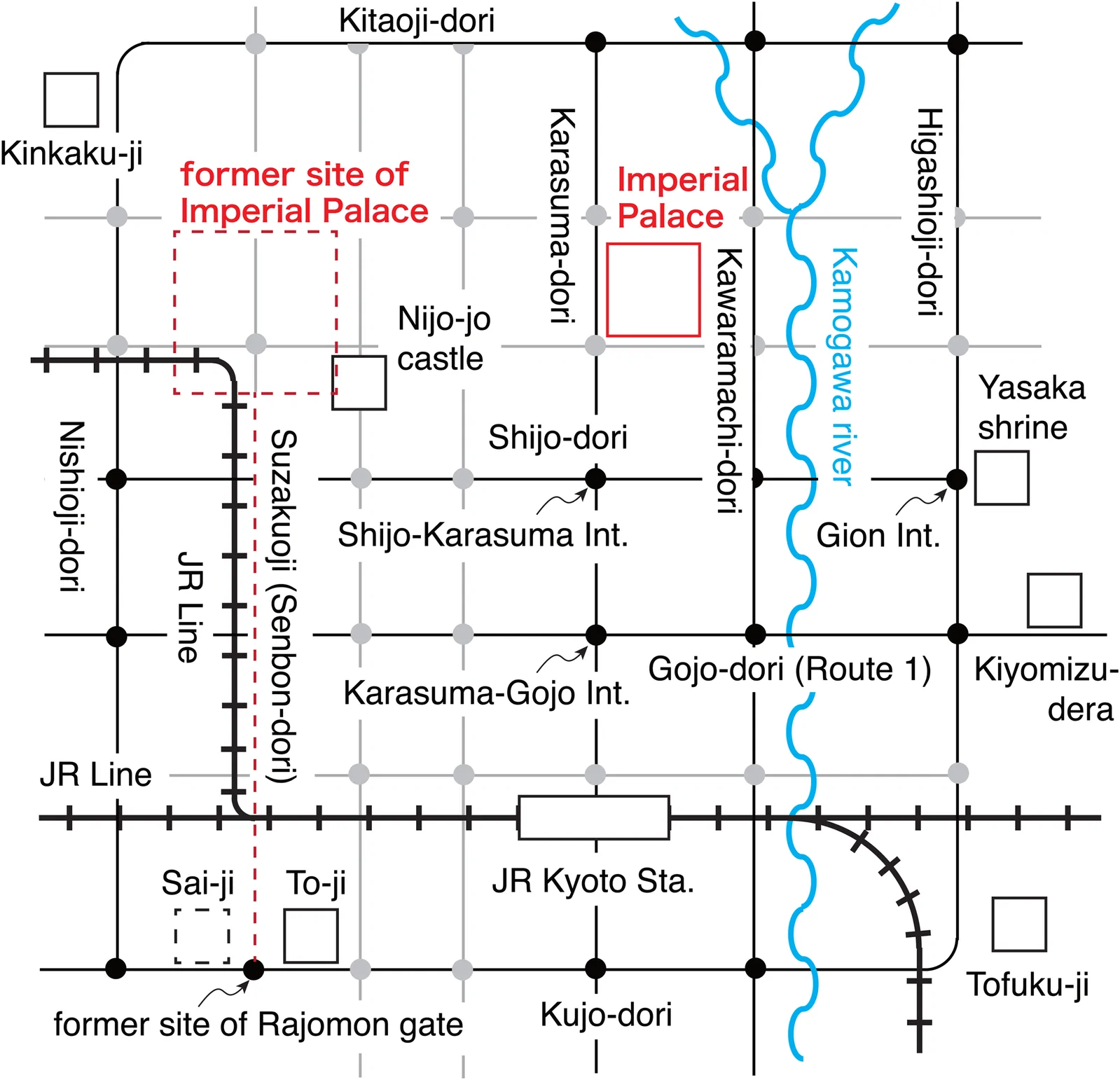
Schematic map of central Kyoto and the streets analyzed in this study. Central Kyoto is bounded by Higashioji-dori, Kujo-dori, Nishioji-dori, and Kitaoji-dori. The Shijo-Karasuma intersection of Shijo-dori, running east–west, and Karasuma-dori, running north–south, sits at the center of Kyoto City.The red solid outline marks the present Imperial Palace and the red dashed outline its approximate Heian-kyo location. The blue line represents the Kamogawa River, and thick lines with crossbars represent railway corridors. River topography and railway lines interrupt the otherwise grid-like network.

In central Kyoto, intersection names are not merely landmarks but also form part of the traditional address system. An intersection can be identified by combining its east–west and north–south street names, as in “Shijo-Karasuma.” A location near that intersection is then specified with a directional term: “Agaru” (going north), “Sagaru” (going south), “Higashi-iru” (entering east), or “Nishi-iru” (entering west). For example, “Shijo-dori Karasuma Nishi-iru” denotes a location on Shijo-dori west of the Karasuma intersection. This differs from the grid system in central Sapporo, where expressions such as “North 3 West 4” identify a block rather than a formally named intersection. The comparison helps clarify why the order and familiarity of combined street names have practical as well as cultural significance in Kyoto. Kyoto’s grid-like layout is a legacy of Heian-kyo, the capital established by Emperor Kanmu at the end of the eighth century. From the Imperial Palace, Suzakuoji extended southward to the Rajomon gate, the main entrance to the capital, with To-ji and Sai-ji temples placed on either side. Heian-kyo was modeled after Chang’an, the capital of Tang dynasty China, and was constructed under the regular urban system known as the Jobo-sei system. The planned city measured approximately 4.5 km from east to west and 5.2 km from north to south. The districts east and west of the palace were designated Sakyo (Left Capital) and Ukyo (Right Capital), respectively. The location of the Imperial Palace later shifted approximately 2 km eastward. The original Suzakuoji corresponds approximately to present-day Senbon-dori, and a stone monument near the Kujo–Kyu-senbon intersection marks the former Rajomon site. Although later urban development, railway construction, and river topography altered parts of the original grid, the basic east–west and north–south organization remains visible in central Kyoto (see Fig 1).

Under the Jobo-sei system, major east–west streets were named Ichijo-oji (First Street) through Kujo-oji (Ninth Street), while east–west positions were divided into First bo through Fourth bo. Heian-period documents identified plots with expressions such as “Sakyo Sanjo Ichibo,” placing the Jo, or east–west coordinate, first [10,12]. The Jo designations survive in present street names from Ichijo-dori through Kujo-dori, whereas the Bo designations gradually disappeared and the north–south streets came to be known by individual names.

Originally, a square block constituted one town unit. As commerce and craft production developed from the Muromachi through Momoyama periods (1336–1603), residents on both sides of a street formed self-governing communities called ryogawa-machi (“both-sides towns”). Communities organized along north–south streets were called tate-machi, whereas those organized along east–west streets were called yoko-machi. Each community tended to place the name of its own street first when referring to the local intersection [12]. Where one community had greater economic or social influence, its preferred order could become dominant. At many intersections, however, both orders remained in use into the Meiji era (1868–1912).

Intersection names were increasingly standardized with the inauguration of the Kyoto City Tram in 1912. Because one stop required one official name, the name of the busier or more familiar street was generally placed first. Although the tram system was abolished in 1978, many stop names remained in use as city-bus stop names. Installation of official intersection signs progressed during the 1970s, and the forms most familiar to residents were adopted [10,11,14]. Consequently, names such as “Shichijo-Kawaramachi” coexist with building or bus-stop names that reverse the order. The present naming network therefore contains traces of the ancient coordinate system, neighborhood organization, transport history, and modern administrative standardization rather than a single uniform rule.

### Street selection and data collection

The dataset contains eight east–west streets (Kitaoji-dori, Imadegawa-dori, Marutamachi-dori, Sanjo-dori, Shijo-dori, Gojo-dori, Shichijo-dori, and Kujo-dori) and eight north–south streets (Nishioji-dori, Onmae-dori, Senbon-dori, Omiya-dori, Horikawa-dori, Karasuma-dori, Kawaramachi-dori, and Higashioji-dori). Oike-dori, beneath which the Tozai (east–west) subway line runs, was not included because it recorded neither a win nor a draw against the selected north–south streets. Its row in the coefficient matrix for the Keener method would therefore be zero, violating the irreducibility condition required by the method. Because this exclusion was based on the observed comparison outcomes, the resulting ranking, particularly its lowest positions, should be interpreted as conditional on the 16 streets included in the analysis.

Intersection names were checked against physical traffic signs and cross-validated using Google Maps and Google Street View. Each intersection between a selected east–west street and a selected north–south street was treated as one pairwise comparison. If the official intersection name contains both street names, the name appearing first receives a win and the name appearing second receives a loss. An intersection name containing neither street name, such as “Gion” at the intersection between Shijo-dori and Higashioji-dori, is treated as a draw. If no intersection name was available for a street pair, the comparison was recorded as missing rather than as a draw. Table 1 reports all comparison outcomes. In total, the dataset contains 51 decisive comparisons, eight draws, and five missing comparisons. Several connected road segments are treated as one street to preserve the functional network shown in Table 1. Shin-senbon-dori and Kyu-senbon-dori are treated as extensions of Senbon-dori. Aburanokoji-dori is treated as an extension of Horikawa-dori south of the JR railway line, while Shimogamo-hondori is considered an extension of Kawaramachi-dori north of Imadegawa-dori. Higashiyama-dori is used for the relevant section of Higashioji-dori. The Tofukuji change point is treated as the Kujo-dori–Higashioji-dori intersection. At the northwestern corner, Nishioji-dori connects to Kitaoji-dori, although the exact point where the street names change has no official intersection name. Approximately 100 m south, a traffic sign at Nishioji-dori and Kuramaguchi-dori bears the name “Kinkakujimae.” This signposted name is used for the Kitaoji-dori–Nishioji-dori comparison in this analysis. The name was formerly used for a tram stop and remains in a nearby bus-stop name.

**Table 1:** Kyoto streets and intersection names. The table summarizes the intersection names of major streets in central Kyoto. The rows represent east–west streets, and the columns represent north–south streets. Each cell lists the corresponding intersection name. An em dash denotes a missing comparison. Based on which street is mentioned first, wins, losses, and draws (W/L/D) are tallied in the final row and column. The data were compiled by verifying physical traffic signs on-site, as well as by cross-referencing intersection labels on Google Maps and confirming them via Google Street View.

Street	Nishioji (Ns)	Onmae (On)	Senbon (Sb)	Omiya (Om)	Horikawa (Hr)	Karasuma (Kr)	Kawaramachi (Kw)	Higashioji (Hg)	W/L/D
Kitaoji (Kt)	Kinkakujimae	—	Senbon-Kitaoji	Kitaoji-Omiya	Horikawa-Kitaoji	Karasuma-Kitaoji	Shimogamo-hondori-Kitaoji	Takano	1/4/2
Imadegawa (Im)	Kitano-hakubaicho	Kitanotenmangu- Shrine	Senbon-Imadegawa	—	Horikawa-Imadegawa	Karasuma-Imadegawa	Kawaramachi-Imadegawa	Hyakumanben	0/4/3
Marutamachi (Mr)	Enmachi	Marutamachi-Onmae	Senbon-Marutamachi	—	Horikawa-Marutamachi	Karasuma-Marutamachi	Kawaramachi-Marutamachi	Higashiyama-Marutamachi	1/5/1
Sanjo (Sn)	Nishioji-Sanjo	Sanjo-Onmae	Senbon-Sanjo	—	Horikawa-Sanjo	Karasuma-Sanjo	Kawaramachi-Sanjo	Higashiyama-Sanjo	1/6/0
Shijo (Sj)	Nishioji-Shijo	Onmaedori-Shijo	—	Shijo-Omiya	Shijo-Horikawa	Shijo-Karasuma	Shijo-Kawaramachi	Gion	4/2/1
Gojo (Gj)	Nishioji-Gojo	Gojo-Onmae	Gojo-Senbon	Omiya-Gojo	Horikawa-Gojo	Karasuma-Gojo	Kawaramachi-Gojo	Higashiyama-Gojo	2/6/0
Shichijo (Sc)	Nishioji-Shichijo	Shichijo-Onmae	Shichijo-Shin-senbon	Omiya-Shichijo	Shichijo-Horikawa	Karasuma-Shichijo	Shichijo-Kawaramachi	Higashiyama-Shichijo	4/4/0
Kujo (Kj)	Nishioji-Kujo	Kujo-Onmae	Kujo-Kyu-senbon	Kujo-Omiya	Kujo-Aburanokoji	Kujo-Karasuma	Kujo-Kawaramachi	Tofukuji	6/1/1
W/L/D	5/0/3	1/5/1	4/3/0	2/3/0	5/3/0	6/2/0	5/3/0	4/0/4	

**Fig 2:**
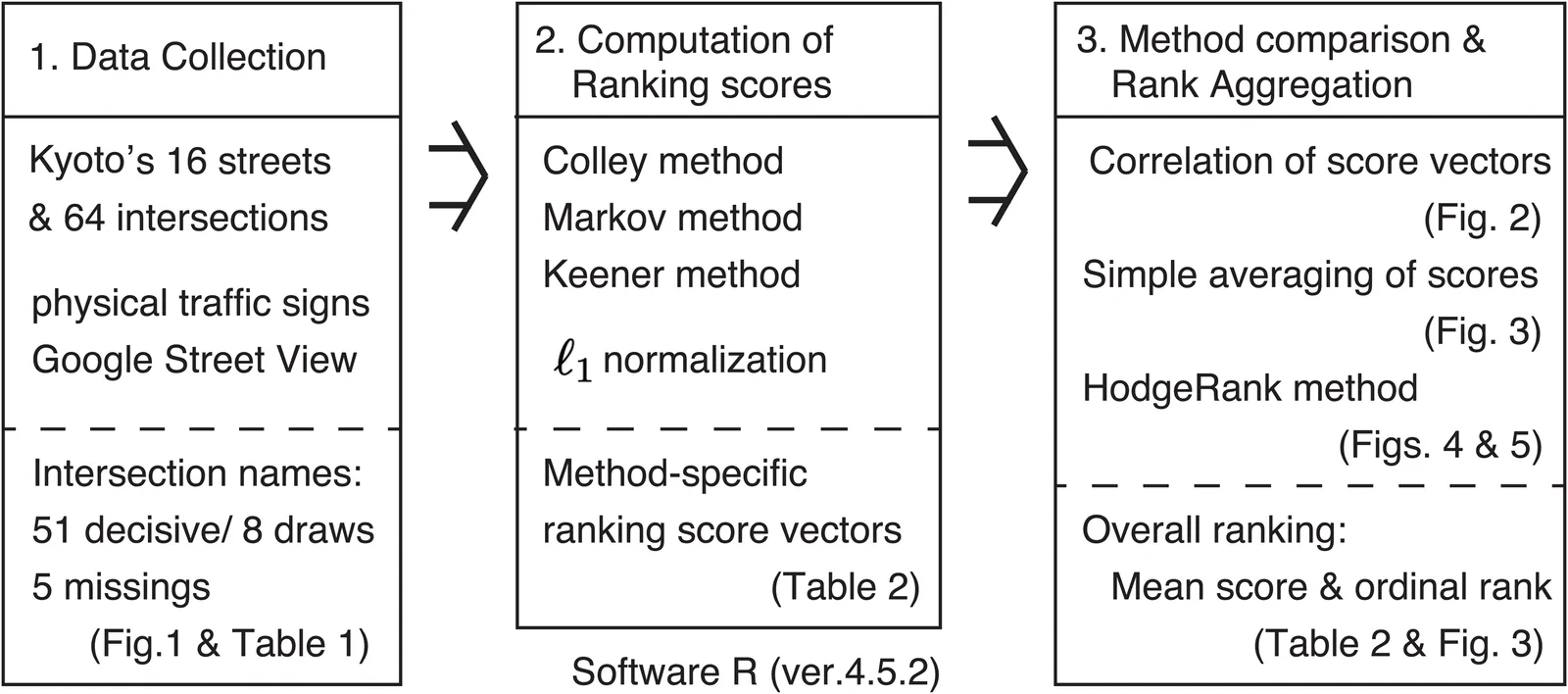
Research workflow. The workflow comprised three stages. (1) Data Collection: selection of 16 streets, verification of 64 possible street pairs from physical traffic signs, Google Maps, and Google Street View, and coding of each pair as a decisive comparison, draw, or missing comparison. (2) Ranking Analysis: computation of Colley, Keener, and Markov chain-based score vectors followed by unit $\ell_{1}$ normalization. (3) Result Assessment: comparison of score and rank correlations, aggregation of normalized scores and ordinal ranks, and HodgeRank assessment of the common gradient and residual disagreement.

The research workflow comprised three stages (Fig 2). In the Data Collection stage, 64 possible pairs were defined from the eight selected east–west and eight selected north–south streets. Intersection names were verified using physical traffic signs, Google Maps, and Google Street View and were coded as decisive outcomes, draws, or missing comparisons according to the rules defined above. This stage yielded 51 decisive comparisons, eight draws, and five missing comparisons. In the Ranking Analysis stage, score vectors were computed independently using the Colley, Keener, and Markov chain-based methods and normalized to have unit $\ell_{1}$ norm. In the Result Assessment stage, the methods were compared using score and rank correlations, normalized scores and ordinal ranks were aggregated, and HodgeRank was used to separate the common ranking gradient from residual disagreement among methods. When two or more streets tied in score, each was assigned the average of the rank positions they spanned. All numerical calculations were performed using R version 4.5.2.

### Winning percentage and Colley’s method

Let $W_{i}$, $L_{i}$, and $D_{i}$ denote the observed numbers of wins, losses, and draws for street *i*, respectively, and let $t_{i}=W_{i}+L_{i}+D_{i}$ be its number of observed comparisons. The preliminary winning percentage is


$$\begin{array}{c} r_{i}=\frac{W_{i}}{W_{i}+L_{i}}. \end{array}$$ (1)

In this preliminary measure, the winning percentage is calculated using only decisive comparisons, with draws excluded. For the data in Table 1, Nishioji-dori and Higashioji-dori both have a winning percentage of 1 because neither has a loss. The measure cannot decide between them, and neither street can be compared directly with the other because both run north–south. Their indirect outcomes also differ: Nishioji-dori defeated Shijo-dori but drew with Kitaoji-dori, Imadegawa-dori, and Marutamachi-dori, whereas Higashioji-dori drew with Shijo-dori and defeated Marutamachi-dori. This example illustrates why win percentage alone is insufficient.

The Colley method was developed in sports data analysis to rank teams when not every team plays every other team [1,2]. For this method, define the adjusted numbers of wins and losses by


$$\begin{array}{c} w_{i}=W_{i}+\frac{D_{i}}{2},\;l_{i}=L_{i}+\frac{D_{i}}{2}. \end{array}$$ (2)

Then $t_{i}=w_{i}+l_{i}$. The method begins with the following modified winning percentage:


$$\begin{array}{c} r_{i}=\frac{w_{i}+1}{t_{i}+2}, \end{array}$$ (3)

which is related to Laplace’s rule of succession. Before any match has been played, Eq. 3 assigns every entity the neutral value 1/2, avoiding the undefined value 0/0 in Eq. 1. After one win it gives 2/3, and after one loss it gives 1/3, rather than the extreme values 1 and 0.

Colley’s opponent adjustment follows from


$$\begin{array}{c} w_{i}=\frac{w_{i}-l_{i}}{2}+\frac{t_{i}}{2}\approx \frac{w_{i}-l_{i}}{2}+\sum_{j\in O_{i}}r_{j}, \end{array}$$ (4)

where $O_{i}$ is the set of observed opponents. The neutral contribution $t_{i}/2$ is replaced by the sum of the opponents’ ranking scores. Substitution into Eq. 3 gives


$$\begin{array}{c} r_{i}=\frac{1+(w_{i}-l_{i})/2+\sum_{j\in O_{i}}r_{j}}{2+t_{i}}. \end{array}$$ (5)

These equations form the linear system


$$\begin{array}{c} C\;r^{Colley}=b, \end{array}$$ (6)

with


$$\begin{array}{c} C_{ij}=\left\{ \begin{array}{cc} 2+t_{i}, & i=j,\\ -\delta_{ij}, & i\neq j, \end{array}\right.\;b_{i}=1+\frac{w_{i}-l_{i}}{2}=1+\frac{W_{i}-L_{i}}{2}. \end{array}$$ (7)

Here, $\delta_{ij}=1$ if streets *i* and *j* have an observed comparison and 0 otherwise. For example, Kujo-dori has six wins, one loss, and one draw, so its adjusted record is *w*_8_ = 13/2 and *l*_8_ = 3/2, giving *b*_8_ = 7/2. For the 16 streets, 1


$$\begin{array}{c} b={\;}^{t}\;\begin{array}{c} \begin{array}{cccccccccccccccc} \text{Kt} & \text{Im} & \text{Mr} & \text{Sn} & \text{Sj} & \text{Gj} & \text{Sc} & \text{Kj} & \text{Ns} & \text{On} & \text{Sb} & \text{Om} & \text{Hr} & \text{Kr} & \text{Kw} & \text{Hg} \end{array}\\ \left(\begin{array}{cccccccccccccccc} -\frac{1}{2} & -1 & -1 & -\frac{3}{2} & 2 & -1 & 1 & \frac{7}{2} & \frac{7}{2} & -1 & \frac{3}{2} & \frac{1}{2} & 2 & 3 & 2 & 3 \end{array}\right) \end{array}. \end{array}$$

The corresponding coefficient matrix *C* is 1


$$\begin{array}{c} C=\;\mathrm{-2.7pc}\begin{array}{c} \begin{array}{cccccccccccccccc} \text{Kt} & \text{Im} & \text{Mr} & \text{Sn} & \text{Sj} & \text{Gj} & \text{Sc} & \text{Kj} & \text{Ns} & \text{On} & \text{Sb} & \text{Om} & \text{Hr} & \text{Kr} & \text{Kw} & \text{Hg} \end{array}\\ \;2pc\left(\begin{array}{cccccccccccccccc} 9 & 0 & 0 & 0 & 0 & 0 & 0 & 0 & -1 & 0 & -1 & -1 & -1 & -1 & -1 & -1\\ 0 & 9 & 0 & 0 & 0 & 0 & 0 & 0 & -1 & -1 & -1 & 0 & -1 & -1 & -1 & -1\\ 0 & 0 & 9 & 0 & 0 & 0 & 0 & 0 & -1 & -1 & -1 & 0 & -1 & -1 & -1 & -1\\ 0 & 0 & 0 & 9 & 0 & 0 & 0 & 0 & -1 & -1 & -1 & 0 & -1 & -1 & -1 & -1\\ 0 & 0 & 0 & 0 & 9 & 0 & 0 & 0 & -1 & -1 & 0 & -1 & -1 & -1 & -1 & -1\\ 0 & 0 & 0 & 0 & 0 & 10 & 0 & 0 & -1 & -1 & -1 & -1 & -1 & -1 & -1 & -1\\ 0 & 0 & 0 & 0 & 0 & 0 & 10 & 0 & -1 & -1 & -1 & -1 & -1 & -1 & -1 & -1\\ 0 & 0 & 0 & 0 & 0 & 0 & 0 & 10 & -1 & -1 & -1 & -1 & -1 & -1 & -1 & -1\\ -1 & -1 & -1 & -1 & -1 & -1 & -1 & -1 & 10 & 0 & 0 & 0 & 0 & 0 & 0 & 0\\ 0 & -1 & -1 & -1 & -1 & -1 & -1 & -1 & 0 & 9 & 0 & 0 & 0 & 0 & 0 & 0\\ -1 & -1 & -1 & -1 & 0 & -1 & -1 & -1 & 0 & 0 & 9 & 0 & 0 & 0 & 0 & 0\\ -1 & 0 & 0 & 0 & -1 & -1 & -1 & -1 & 0 & 0 & 0 & 7 & 0 & 0 & 0 & 0\\ -1 & -1 & -1 & -1 & -1 & -1 & -1 & -1 & 0 & 0 & 0 & 0 & 10 & 0 & 0 & 0\\ -1 & -1 & -1 & -1 & -1 & -1 & -1 & -1 & 0 & 0 & 0 & 0 & 0 & 10 & 0 & 0\\ -1 & -1 & -1 & -1 & -1 & -1 & -1 & -1 & 0 & 0 & 0 & 0 & 0 & 0 & 10 & 0\\ -1 & -1 & -1 & -1 & -1 & -1 & -1 & -1 & 0 & 0 & 0 & 0 & 0 & 0 & 0 & 10 \end{array}\right)\begin{array}{c} \text{Kt}\\ \text{Im}\\ \text{Mr}\\ \text{Sn}\\ \text{Sj}\\ \text{Gj}\\ \text{Sc}\\ \text{Kj}\\ \text{Ns}\\ \text{On}\\ \text{Sb}\\ \text{Om}\\ \text{Hr}\\ \text{Kr}\\ \text{Kw}\\ \text{Hg} \end{array}. \end{array} \end{array}$$

Solving Eq. 6 gives the Colley scores reported in Table 2. The solution was rescaled so that $\|r^{Colley}{\|}_{1}=1$.

**Table 2:** Normalized ranking scores. Ordinal ranks are given in parentheses. Ranks were assigned in descending order of score, with rank 1 corresponding to the largest score. Ties were assigned midranks. The final column shows the simple average of the three normalized scores for each street.

*i*	Street	$r_{i}^{Colley}$	$r_{i}^{Keener}$	$r_{i}^{Markov}$	${\overset{―}{r}}_{i}$
1	Kitaoji (Kt)	0.05031 (11)	0.05394 (9)	0.08000 (5)	0.06142 (6)
2	Imadegawa (Im)	0.04042 (13.5)	0.04519 (12)	0.07836 (6)	0.05466 (10)
3	Marutamachi (Mr)	0.04042 (13.5)	0.03631 (14)	0.05754 (8)	0.04476 (12)
4	Sanjo (Sn)	0.03348 (15)	0.01506 (16)	0.00785 (16)	0.01880 (16)
5	Shijo (Sj)	0.08156 (5)	0.09845 (3)	0.09012 (4)	0.09004 (4)
6	Gojo (Gj)	0.04212 (12)	0.02956 (15)	0.02186 (14)	0.03118 (15)
7	Shichijo (Sc)	0.06712 (8)	0.06387 (6)	0.05230 (9)	0.06110 (7)
8	Kujo (Kj)	0.09837 (1)	0.11571 (2)	0.11198 (2)	0.10869 (2)
9	Nishioji (Ns)	0.08913 (2)	0.12049 (1)	0.14908 (1)	0.11957 (1)
10	Onmae (On)	0.03094 (16)	0.04270 (13)	0.04317 (12)	0.03894 (14)
11	Senbon (Sb)	0.06219 (9)	0.05309 (10)	0.04202 (13)	0.05243 (11)
12	Omiya (Om)	0.05743 (10)	0.04614 (11)	0.01672 (15)	0.04009 (13)
13	Horikawa (Hr)	0.07038 (6.5)	0.05558 (7.5)	0.04566 (10.5)	0.05721 (8.5)
14	Karasuma (Kr)	0.08288 (3.5)	0.07529 (5)	0.05873 (7)	0.07230 (5)
15	Kawaramachi (Kw)	0.07038 (6.5)	0.05558 (7.5)	0.04566 (10.5)	0.05721 (8.5)
16	Higashioji (Hg)	0.08288 (3.5)	0.09304 (4)	0.09896 (3)	0.09163 (3)

### Eigenvalues and the Keener method

In the Colley method, the combinations of comparisons are represented by the coefficient matrix *C*, while the right-hand side vector b is determined by the net win–loss differences. Consequently, the method evaluates the strength of a street’s opponents as an aggregate but is less sensitive to the identity of the opponent defeated in a particular comparison. The Keener method addresses this issue by giving a favorable outcome greater value when it is obtained against a highly rated opponent [3].

Define the score of street *i* by


$$\begin{array}{c} p_{i}=\frac{1}{t_{i}}\sum_{j\neq i}a_{ij}r_{j}, \end{array}$$ (8)

where $a_{ij}=1$ if street *i* defeats street *j*, $a_{ij}=1/2$ for a draw, and $a_{ij}=0$ for a loss or missing comparison. Thus, $a_{ij}+a_{ji}=1$ whenever an observed comparison occurs. Dividing by $t_{i}$ prevents streets with more observed comparisons from receiving a larger score solely for that reason. The Keener self-consistency condition requires a high score $p_{i}$ to correspond to a high ranking score $r_{i}$, so $p_{i}=\lambda r_{i}$. With $A_{ij}=a_{ij}/t_{i}$, this becomes


$$\begin{array}{c} A\;r^{Keener}=\lambda_{\max}r^{Keener}. \end{array}$$ (9)

The Perron–Frobenius theorem guarantees that an irreducible non-negative matrix has a positive maximum eigenvalue and a corresponding strictly positive eigenvector, unique up to multiplication by a scalar. In the ranking context, irreducibility means that every street is connected to every other street through directed outcomes, possibly by a chain of intermediate streets. A street with only losses would produce a zero row. This is the reason for excluding Oike-dori from the calculation by the Keener method.

For the present data, the coefficient matrix *A* is 1


$$\begin{array}{c} A=\;\mathrm{-2.7pc}\begin{array}{c} \begin{array}{cccccccccccccccc} \text{Kt} & \text{Im} & \text{Mr} & \text{Sn} & \text{Sj} & \text{Gj} & \text{Sc} & \text{Kj} & \text{Ns} & \text{On} & \text{Sb} & \text{Om} & \text{Hr} & \text{Kr} & \text{Kw} & \text{Hg} \end{array}\\ \;2pc\left(\begin{array}{cccccccccccccccc} 0 & 0 & 0 & 0 & 0 & 0 & 0 & 0 & \frac{1}{14} & 0 & 0 & \frac{1}{7} & 0 & 0 & 0 & \frac{1}{14}\\ 0 & 0 & 0 & 0 & 0 & 0 & 0 & 0 & \frac{1}{14} & \frac{1}{14} & 0 & 0 & 0 & 0 & 0 & \frac{1}{14}\\ 0 & 0 & 0 & 0 & 0 & 0 & 0 & 0 & \frac{1}{14} & \frac{1}{7} & 0 & 0 & 0 & 0 & 0 & 0\\ 0 & 0 & 0 & 0 & 0 & 0 & 0 & 0 & 0 & \frac{1}{7} & 0 & 0 & 0 & 0 & 0 & 0\\ 0 & 0 & 0 & 0 & 0 & 0 & 0 & 0 & 0 & 0 & 0 & \frac{1}{7} & \frac{1}{7} & \frac{1}{7} & \frac{1}{7} & \frac{1}{14}\\ 0 & 0 & 0 & 0 & 0 & 0 & 0 & 0 & 0 & \frac{1}{8} & \frac{1}{8} & 0 & 0 & 0 & 0 & 0\\ 0 & 0 & 0 & 0 & 0 & 0 & 0 & 0 & 0 & \frac{1}{8} & \frac{1}{8} & 0 & \frac{1}{8} & 0 & \frac{1}{8} & 0\\ 0 & 0 & 0 & 0 & 0 & 0 & 0 & 0 & 0 & \frac{1}{8} & \frac{1}{8} & \frac{1}{8} & \frac{1}{8} & \frac{1}{8} & \frac{1}{8} & \frac{1}{16}\\ \frac{1}{16} & \frac{1}{16} & \frac{1}{16} & \frac{1}{8} & \frac{1}{8} & \frac{1}{8} & \frac{1}{8} & \frac{1}{8} & 0 & 0 & 0 & 0 & 0 & 0 & 0 & 0\\ 0 & \frac{1}{14} & 0 & 0 & \frac{1}{7} & 0 & 0 & 0 & 0 & 0 & 0 & 0 & 0 & 0 & 0 & 0\\ \frac{1}{7} & \frac{1}{7} & \frac{1}{7} & \frac{1}{7} & 0 & 0 & 0 & 0 & 0 & 0 & 0 & 0 & 0 & 0 & 0 & 0\\ 0 & 0 & 0 & 0 & 0 & \frac{1}{5} & \frac{1}{5} & 0 & 0 & 0 & 0 & 0 & 0 & 0 & 0 & 0\\ \frac{1}{8} & \frac{1}{8} & \frac{1}{8} & \frac{1}{8} & 0 & \frac{1}{8} & 0 & 0 & 0 & 0 & 0 & 0 & 0 & 0 & 0 & 0\\ \frac{1}{8} & \frac{1}{8} & \frac{1}{8} & \frac{1}{8} & 0 & \frac{1}{8} & \frac{1}{8} & 0 & 0 & 0 & 0 & 0 & 0 & 0 & 0 & 0\\ \frac{1}{8} & \frac{1}{8} & \frac{1}{8} & \frac{1}{8} & 0 & \frac{1}{8} & 0 & 0 & 0 & 0 & 0 & 0 & 0 & 0 & 0 & 0\\ \frac{1}{16} & \frac{1}{16} & \frac{1}{8} & \frac{1}{8} & \frac{1}{16} & \frac{1}{8} & \frac{1}{8} & \frac{1}{16} & 0 & 0 & 0 & 0 & 0 & 0 & 0 & 0 \end{array}\right)\begin{array}{c} \text{Kt}\\ \text{Im}\\ \text{Mr}\\ \text{Sn}\\ \text{Sj}\\ \text{Gj}\\ \text{Sc}\\ \text{Kj}\\ \text{Ns}\\ \text{On}\\ \text{Sb}\\ \text{Om}\\ \text{Hr}\\ \text{Kr}\\ \text{Kw}\\ \text{Hg} \end{array} \end{array} \end{array}$$

The matrix is irreducible and has Perron–Frobenius eigenvalue $\lambda_{\max}\approx 0.40499$. Because the comparison structure is bipartite, repeated multiplication by *A* may alternate rather than converge, even though the positive Perron eigenvector is well defined. We therefore solve the eigenvalue problem in Eq. 9 directly for the positive eigenvector and normalize it to $\|r^{Keener}{\|}_{1}=1$. The resulting scores are reported in Table 2.

### Markov chain-based method

The third method is inspired by the voting interpretation of PageRank [4,15]. In a web network, a page receives authority from pages that link to it, and the weight of an outgoing vote is divided among its targets. Repeated movement according to these link probabilities produces a Markov chain whose equilibrium distribution represents long-run authority. In the present application, a losing street transfers weight to the street that defeated it, whereas a draw transfers half weight in each direction.

Specifically, define $s_{ij}=1$ if street *i* loses to street *j*, $s_{ij}=1/2$ for a draw, and $s_{ij}=0$ for a win or missing comparison. The transition probability matrix *S* is


$$\begin{array}{c} S_{ij}=\frac{s_{ij}}{\sum_{h}s_{ih}}. \end{array}$$ (10)

Thus, every street distributes one unit of transition probability among the streets to which it lost or drew. If a row has no positive outgoing weight, it is replaced by the uniform distribution $S_{ij}=1/n$, analogously to the treatment of a dangling node in PageRank. The entries satisfy $0\leq S_{ij}\leq 1$ and $\sum_{j}S_{ij}=1$.

For the intersection data, the transition probability matrix *S* is 1


$$\begin{array}{c} S=\begin{array}{c} \begin{array}{cccccccccccccccc} \text{Kt} & \text{Im} & \text{Mr} & \text{Sn} & \text{Sj} & \text{Gj} & \text{Sc} & \text{Kj} & \text{Ns} & \text{On} & \text{Sb} & \text{Om} & \text{Hr} & \text{Kr} & \text{Kw} & \text{Hg} \end{array}\\ \;2pc\left(\begin{array}{cccccccccccccccc} 0 & 0 & 0 & 0 & 0 & 0 & 0 & 0 & \frac{1}{10} & 0 & \frac{1}{5} & 0 & \frac{1}{5} & \frac{1}{5} & \frac{1}{5} & \frac{1}{10}\\ 0 & 0 & 0 & 0 & 0 & 0 & 0 & 0 & \frac{1}{11} & \frac{1}{11} & \frac{2}{11} & 0 & \frac{2}{11} & \frac{2}{11} & \frac{2}{11} & \frac{1}{11}\\ 0 & 0 & 0 & 0 & 0 & 0 & 0 & 0 & \frac{1}{11} & 0 & \frac{2}{11} & 0 & \frac{2}{11} & \frac{2}{11} & \frac{2}{11} & \frac{2}{11}\\ 0 & 0 & 0 & 0 & 0 & 0 & 0 & 0 & \frac{1}{6} & 0 & \frac{1}{6} & 0 & \frac{1}{6} & \frac{1}{6} & \frac{1}{6} & \frac{1}{6}\\ 0 & 0 & 0 & 0 & 0 & 0 & 0 & 0 & \frac{2}{5} & \frac{2}{5} & 0 & 0 & 0 & 0 & 0 & \frac{1}{5}\\ 0 & 0 & 0 & 0 & 0 & 0 & 0 & 0 & \frac{1}{6} & 0 & 0 & \frac{1}{6} & \frac{1}{6} & \frac{1}{6} & \frac{1}{6} & \frac{1}{6}\\ 0 & 0 & 0 & 0 & 0 & 0 & 0 & 0 & \frac{1}{4} & 0 & 0 & \frac{1}{4} & 0 & \frac{1}{4} & 0 & \frac{1}{4}\\ 0 & 0 & 0 & 0 & 0 & 0 & 0 & 0 & \frac{2}{3} & 0 & 0 & 0 & 0 & 0 & 0 & \frac{1}{3}\\ \frac{1}{3} & \frac{1}{3} & \frac{1}{3} & 0 & 0 & 0 & 0 & 0 & 0 & 0 & 0 & 0 & 0 & 0 & 0 & 0\\ 0 & \frac{1}{11} & \frac{2}{11} & \frac{2}{11} & 0 & \frac{2}{11} & \frac{2}{11} & \frac{2}{11} & 0 & 0 & 0 & 0 & 0 & 0 & 0 & 0\\ 0 & 0 & 0 & 0 & 0 & \frac{1}{3} & \frac{1}{3} & \frac{1}{3} & 0 & 0 & 0 & 0 & 0 & 0 & 0 & 0\\ \frac{1}{3} & 0 & 0 & 0 & \frac{1}{3} & 0 & 0 & \frac{1}{3} & 0 & 0 & 0 & 0 & 0 & 0 & 0 & 0\\ 0 & 0 & 0 & 0 & \frac{1}{3} & 0 & \frac{1}{3} & \frac{1}{3} & 0 & 0 & 0 & 0 & 0 & 0 & 0 & 0\\ 0 & 0 & 0 & 0 & \frac{1}{2} & 0 & 0 & \frac{1}{2} & 0 & 0 & 0 & 0 & 0 & 0 & 0 & 0\\ 0 & 0 & 0 & 0 & \frac{1}{3} & 0 & \frac{1}{3} & \frac{1}{3} & 0 & 0 & 0 & 0 & 0 & 0 & 0 & 0\\ \frac{1}{4} & \frac{1}{4} & 0 & 0 & \frac{1}{4} & 0 & 0 & \frac{1}{4} & 0 & 0 & 0 & 0 & 0 & 0 & 0 & 0 \end{array}\right)\begin{array}{c} \text{Kt}\\ \text{Im}\\ \text{Mr}\\ \text{Sn}\\ \text{Sj}\\ \text{Gj}\\ \text{Sc}\\ \text{Kj}\\ \text{Ns}\\ \text{On}\\ \text{Sb}\\ \text{Om}\\ \text{Hr}\\ \text{Kr}\\ \text{Kw}\\ \text{Hg} \end{array}. \end{array} \end{array}$$

For example, Gojo-dori lost to six streets, so each of those six receives transition probability 1/6 from the Gojo row. Nishioji-dori had no loss but drew with three streets. After normalization, each of those draws receives probability 1/3. For an initial probability vector $x_{0}$, the Markov update is $x_{m+1}={\;}^{t}\;Sx_{m}$. Because the comparison network is bipartite, repeated updates may alternate rather than converge. We therefore solve directly for the normalized stationary ranking vector satisfying


$$\begin{array}{c} r^{Markov}={\;}^{t}\;S\;r^{Markov},\;\|r^{Markov}{\|}_{1}=1. \end{array}$$ (11)

The ranking scores at equilibrium are given in Table 2. This construction is PageRank-inspired but does not reproduce every component of the web-search algorithm. It uses the transition principle appropriate to the street-comparison data.

### Rank aggregation and HodgeRank analysis

The primary overall ranking vector is the simple average of the three normalized vectors:


$$\begin{array}{c} \overset{―}{r}=\frac{r^{Colley}+r^{Keener}+r^{Markov}}{3}. \end{array}$$ (12)

All three ranking vectors satisfy the normalization condition $\|r^{k}{\|}_{1}=1$, but this does not by itself prove that their score intervals have identical interpretations. We therefore also averaged the ordinal ranks from the three methods as a sensitivity analysis. Agreement was summarized by Pearson correlations of the normalized scores and Spearman correlations of the ordinal ranks.

Next, we apply the Hodge decomposition-based method to express the common ranking component and quantify disagreement among the three methods. A simple average alone does not show where the methods disagree. Let $r_{i}^{k}$ denote the normalized score for street *i* under method k∈{Colley,Keener,Markov} and define one edge-flow observation for each method and unordered pair:


$$\begin{array}{c} Y_{ij}^{k}=r_{i}^{k}-r_{j}^{k},\;i<j. \end{array}$$ (13)

The three sets of pairwise score differences, one for each method, can be regarded as parallel observations for every pair of streets. A common overall ranking vector s is estimated by


$$\begin{array}{c} s^{*}=\underset{\sum_{i}s_{i}=0}{arg\;min}\sum_{k=1}^{3}\sum_{i<j}{\left(s_{i}-s_{j}-Y_{ij}^{k}\right)}^{2}. \end{array}$$ (14)

On this complete graph,


$$\begin{array}{c} s^{*}=\overset{―}{r}-\frac{1}{n}\;1, \end{array}$$ (15)

because each normalized vector sums to one. Thus, the Hodge decomposition gives the same ordering as the arithmetic mean in Eq. 12. Its additional role is to quantify the variation among the three methods [9]. Let ${\hat{Y}}_{ij}=s_{i}^{*}-s_{j}^{*}$ and $\varepsilon_{ij}^{k}=Y_{ij}^{k}-{\hat{Y}}_{ij}$. Orthogonality of the least-squares projection yields


$$\begin{array}{c} E_{total}=E_{gradient}+E_{residual}, \end{array}$$ (16)

where


$$\begin{array}{cc} E_{total} & =\sum_{k=1}^{3}\sum_{i<j}(Y_{ij}^{k}{)}^{2},\;5mmE_{gradient}=\sum_{k=1}^{3}\sum_{i<j}{\hat{Y}}_{ij}^{\;2},\;5mmE_{residual}=\sum_{k=1}^{3}\sum_{i<j}(\varepsilon_{ij}^{k}{)}^{2}. \end{array}$$ (17)

Because every $Y^{k}$ is itself generated by an overall ranking vector, the residuals represent disagreement among the three methods rather than cyclic preferences within any single method or within the raw intersection outcomes. Pair-specific residual magnitude was summarized by $d_{ij}=(\sum_{k}(\varepsilon_{ij}^{k}{)}^{2}{)}^{1/2}$.

## Results

### Descriptive comparison data

The eight east–west streets recorded 19 wins, 32 losses, and eight draws. The north–south streets recorded the reverse win–loss totals. The north–south naming convention therefore appeared first in 32 of the 51 decisive names. Kujo-dori and Karasuma-dori each recorded six wins. Shijo-dori recorded four wins, two losses, and one draw, while Shichijo-dori recorded four wins and four losses. Nishioji-dori and Higashioji-dori had no losses, so a simple winning percentage would place both at 100% despite their different opponents and outcomes.

### Rankings obtained by the three methods

Table 2 reports all normalized scores and ranks. Colley ranked Kujo-dori first and Nishioji-dori second. Keener and Markov both ranked Nishioji-dori first and Kujo-dori second. Shijo-dori was fifth under Colley, third under Keener, and fourth under Markov. Thus, the leading pair was stable as a set, but its internal order depended on the method.

Under the Colley method, Karasuma-dori and Higashioji-dori shared the next score after Kujo-dori and Nishioji-dori, while Shijo-dori was fifth. Sanjo-dori and Onmae-dori occupied the bottom two positions. An instructive feature is that Kujo-dori, the Colley leader, lost its direct comparison with second-ranked Nishioji-dori. Colley nevertheless preferred Kujo-dori because it combined six wins with comparatively strong opponents. The average rank was 7.125 for the eight north–south streets and 9.875 for the eight east–west streets. This directional gap was much smaller than the raw difference of 32 versus 19 wins because Colley adjusted for the strength of the opponents as an aggregate.

The Keener method’s recursive opponent weighting changed the order. Nishioji-dori moved to first, Kujo-dori was second, and Shijo-dori rose to third, followed by Higashioji-dori and Karasuma-dori. Sanjo-dori and Gojo-dori were the bottom two. The average ranks were 7.375 for north–south streets and 9.625 for east–west streets. Compared with Colley, Keener gave greater weight to the identity of the street defeated and therefore rewarded Nishioji-dori’s favorable outcomes against highly rated streets.

The Markov method again ranked Nishioji-dori first, Kujo-dori second, Higashioji-dori third, and Shijo-dori fourth. Kitaoji-dori and Imadegawa-dori rose to fifth and sixth, respectively, and Marutamachi-dori rose to eighth. These increases are consistent with the transition weights assigned to their draws with Nishioji-dori. Markov produced the smallest directional difference: the average ranks were 9.0 for north–south streets and 8.0 for east–west streets.

**Fig 3:**
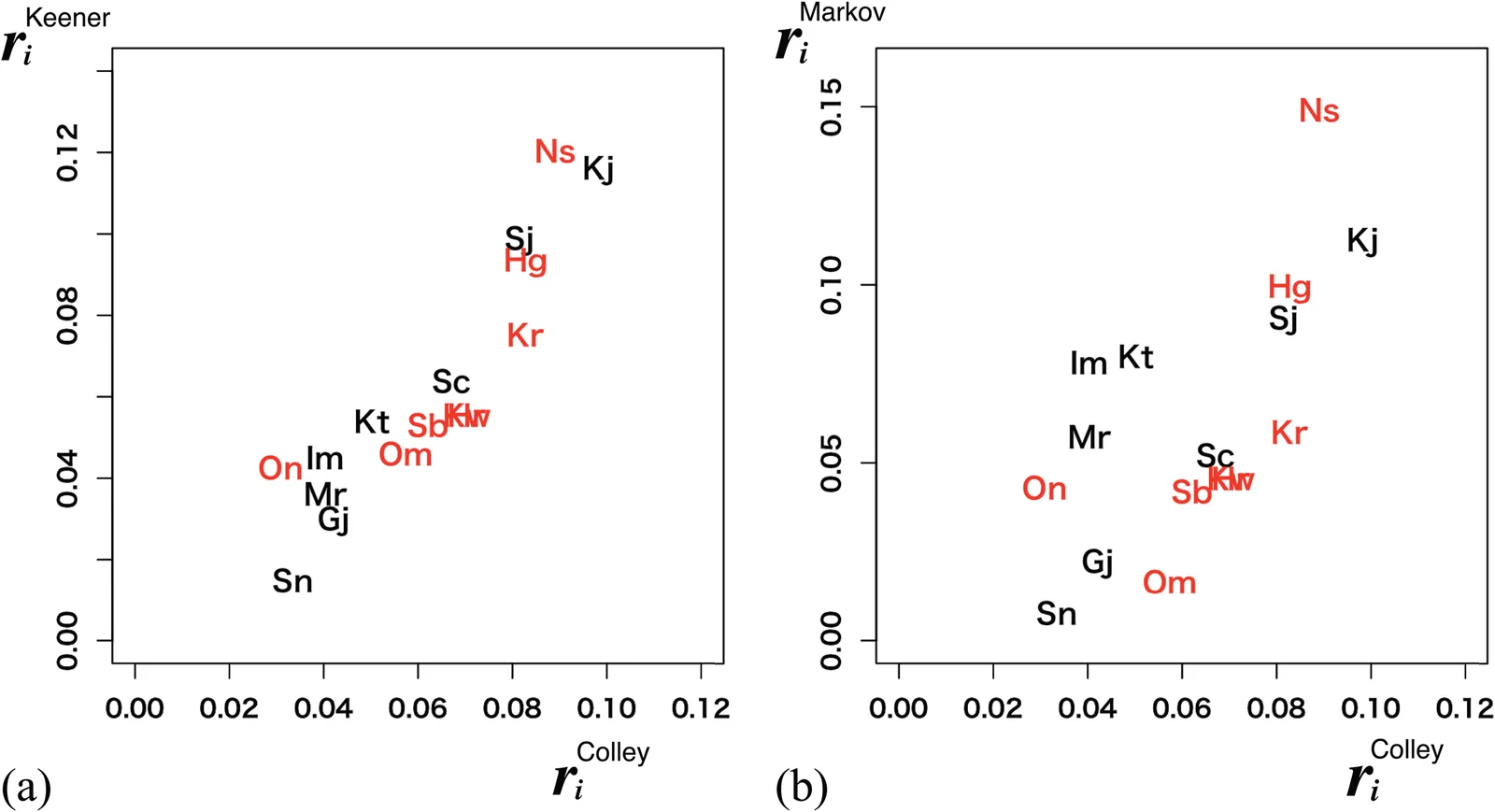
Comparison of normalized ranking scores. The horizontal axis gives Colley scores, and the vertical axis gives (a) Keener scores and (b) Markov scores. Initials of the eight east–west streets are plotted in black and those of the eight north–south streets in red. Horikawa-dori (Hr) and Kawaramachi-dori (Kw) have identical scores and overlap.

The score scatter plots in Fig 3 show that Colley and Keener were most similar. Pearson score correlations were 0.9058 for Colley–Keener, 0.6558 for Colley–Markov, and 0.8776 for Keener–Markov (the corresponding Spearman rank correlations were 0.9381, 0.6563, and 0.7820). Thus, Keener occupied an intermediate position: its normalized scores were close to Colley, while its top-ranked street agreed with Markov.

### Rank aggregation and HodgeRank results

**Fig 4:**
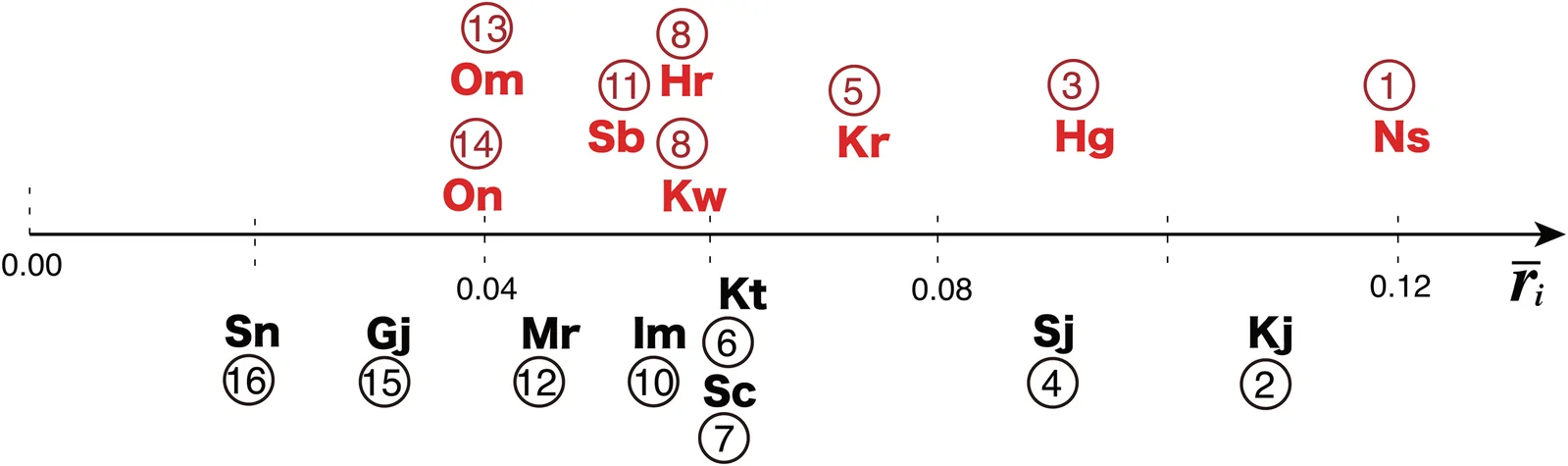
Scores obtained by simple averaging of the Colley, Keener, and Markov results. East–west streets are shown in black and north–south streets in red. Circled numbers indicate the final ranks.

The simple average placed Nishioji-dori first (0.11957), Kujo-dori second (0.10869), Higashioji-dori third (0.09163), and Shijo-dori fourth (0.09004). The small difference between the third and fourth scores indicates that their exact separation should not be overinterpreted. Karasuma-dori ranked fifth, while Sanjo-dori ranked last among the 16 included streets. Fig 4 displays the full score distribution. Averaging ordinal ranks rather than normalized scores gave the same top four: Nishioji-dori (mean rank 4/3), Kujo-dori (5/3), Higashioji-dori (7/2), and Shijo-dori (4). The leading group is therefore robust to this change of aggregation scale, although several middle and lower positions differ.

The total edge-flow energy was $E_{total}=0.6623$. At $s=s^{*}$, the common gradient component accounted for $E_{gradient}=0.5557$ (83.91%) of the total, while the residual sum of squares in Eq. 14 attained its minimum value of $E_{residual}=0.1066$ (16.09%).

**Fig 5:**
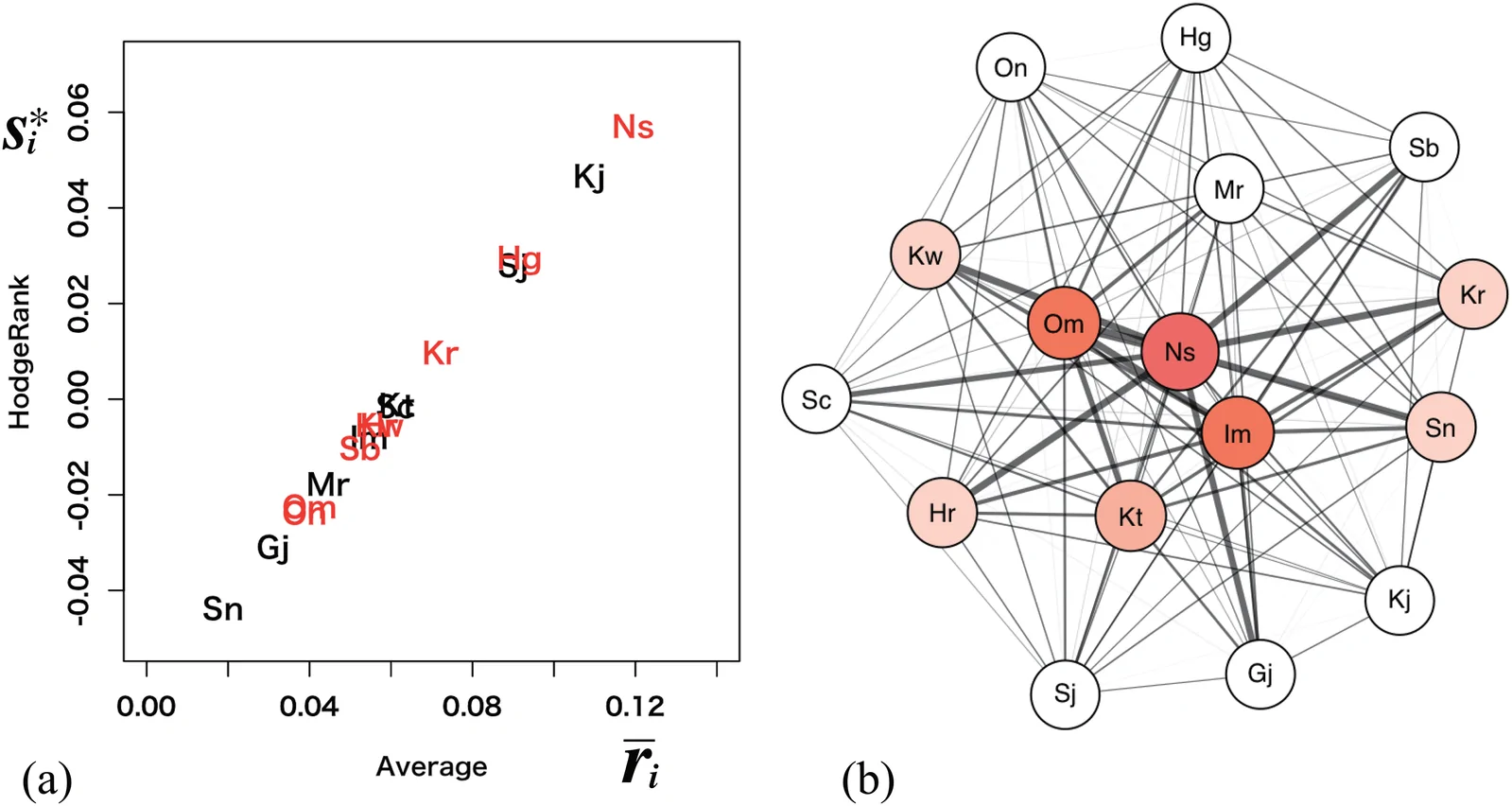
Overall ranking and disagreement among methods. (a) The horizontal axis is the arithmetic mean score $\overset{―}{r}$ and the vertical axis is the centered Hodge score vector $s^{*}$. Their exact affine relationship explains the perfect correlation. (b) Edge thickness represents the residual magnitude $d_{ij}$ across the three methods. Deeper red nodes make larger contributions to the residual sum of squares.

Fig 5(a) confirms the exact affine relationship between the centered Hodge score and the arithmetic mean. Fig 5(b) maps the pairwise residual magnitudes $d_{ij}$. The largest residual contributions were concentrated around Nishioji-dori. Omiya-dori, Imadegawa-dori, and Kitaoji-dori also made relatively large contributions. This pattern means that the methods differed most strongly in the score differences involving these streets. These residuals represent disagreement among the three ranking methods, not an intransitive cycle within any single method.

## Discussion

### Main findings

The main result is not merely that Nishioji-dori receives the highest aggregate score. Table 2 shows that Colley ranked Kujo-dori first and Nishioji-dori second, whereas Keener and Markov reversed that order. Nevertheless, both score averaging and rank averaging yielded the same top four streets: Nishioji-dori, Kujo-dori, Higashioji-dori, and Shijo-dori (Table 2 and Fig 4). Thus, the incomplete bipartite comparison network produced a stable leading group even though the ranking methods disagreed on its internal ordering. This combination of agreement and disagreement is more informative than the result of any single method.

The three methods differ in how they use direct outcomes and transmit their effects through chains of opponents. Colley regularizes the aggregate win–loss balance, Keener recursively values outcomes against highly rated opponents, and Markov is more sensitive to specific comparison paths. Consistent with these differences, the score comparisons in Fig 3 show a higher Pearson correlation for Colley–Keener (0.9058) than for Colley–Markov (0.6558). The residual network in Fig 5 further shows that disagreement was concentrated around particular streets rather than distributed uniformly. The analysis therefore does not identify one universally superior method; rather, it distinguishes conclusions that are robust to method choice from those that remain method-dependent.

### Ranking Kyoto streets from intersection names

Under the operational rule used here, Nishioji-dori ranks first in the overall ranking rather than Shijo-dori. This statement concerns only name precedence. It does not imply that Nishioji-dori is more central, historically important, commercially active, or culturally representative. Indeed, Shijo-dori remains a primary symbolic and commercial axis of central Kyoto. The result instead reveals how the pattern of direct and indirect naming comparisons favors different streets under distinct mathematical formulations.

The contrast that motivated the original question in the newspaper remains important for the interpretation. The area from Shijo-Karasuma to Shijo-Kawaramachi contains major offices, banks, department stores, and shopping districts. Shijo-dori then crosses the Shijo-Ohashi bridge toward Yasaka Shrine at the Gion intersection (see Fig 1). Gojo-dori, by contrast, carries the National Route 1 as far as Horikawa-Gojo and is a wide, heavily trafficked corridor lined with hotels and offices. The historical and contemporary prominence of both streets makes the inconsistent ordering of their intersection names intuitively striking, but those urban qualities are not variables in the present ranking calculation [13].

The imbalance between east–west and north–south wins is also historically suggestive. East–west coordinates were placed first under the early Jōbō-sei system, whereas later neighborhood and transportation practices often favored north–south street names. North–south streets won 32 of the 51 decisive comparisons, compared with 19 wins for east–west streets. The mean ranks of north–south versus east–west streets were 7.125 versus 9.875 under Colley and 7.375 versus 9.625 under Keener. The Markov model reversed this directional difference, yielding mean ranks of 9.0 versus 8.0, because it placed greater weight on the specific comparison paths associated with wins and draws. Thus, the raw 32–19 imbalance did not translate mechanically into the final ranking.

The numerical order should not replace the cultural meanings attached to individual streets. Sanjo-dori ranked last among the 16 included streets, yet historically it served as Kyoto’s gateway at the western terminus of the Tokaido highway connecting Kyoto with Edo. Nishioji-dori, on the contrary, was developed in the early Showa era alongside the Kyoto City Tram and has the shortest history among the selected streets. The three highest-ranked streets—Nishioji-dori, Kujo-dori, and Higashioji-dori—also formed major parts of the former tram loop around central Kyoto [13,14]. These contrasts demonstrate why the operational “strength” measured here is distinct from historical importance or public familiarity.

The purpose of the analysis is therefore not to impose a single, uniquely correct answer on a cultural question. Kyoto’s place names preserve layers of political, commercial, neighborhood, and transport history, and the coexistence of competing name orders is part of their interest. Mathematical ranking adds another perspective to this discussion and provides a way to examine indirect comparisons that are difficult to see from win–loss totals alone.

### General relevance and data journalism

Kyoto intersection names offer a compact and interpretable case study for a broader data-science problem. Similar structures arise whenever comparisons are incomplete, constrained by group membership, or evaluated by several methods. The workflow shown in Fig 2—explicit comparison rules, multiple ranking methods, rank aggregation, and HodgeRank-based analysis—can be transferred to sports analytics, institutional benchmarking, recommendation systems, and preference data.

Data journalism is commonly distinguished from related quantitative forms of journalism by the central role of structured data in discovering, analyzing, and communicating stories [16,17]. In this study, the journalistic question did more than merely provide a colorful example; it defined a publicly understandable problem that could be translated into a structured dataset. Conversely, the statistical analysis re-entered the public sphere through subsequent newspaper reporting [13,18]. This project thus illustrates a two-way pathway between journalism and research: a locally meaningful question motivates systematic data construction, and the resulting analysis is translated back into an accessible narrative for a non-specialist audience.

The relevance of Kyoto’s intersection names is practical as well as analytical. A block-numbering system such as that used in Sapporo may initially be easier for a temporary visitor, whereas navigating Kyoto requires learning how combined street names and directional terms such as “Agaru,” “Sagaru,” “Higashi-iru,” and “Nishi-iru” specify a location. Deciphering this distinctive address system is not only part of the experience of visiting Kyoto. It is also among the first pieces of local knowledge that people beginning a new life in the city for study or work must learn. This helps explain why local media publish accessible articles about the mysteries of Kyoto’s place names for visitors and newcomers [10,11,13].

For over a millennium (794–1868), Kyoto served as the imperial capital of Japan and remains its spiritual and cultural heart. Street and intersection names recall emperors, court nobles, ancient government offices, and other names encountered in Japanese history, thereby connecting the present city with its long past [10,12]. Residents may have a favorite street or a strong preference for the familiar order of an intersection name. Questions about which street name should come first therefore invite discussion not only about navigation but also about neighborhood identity, historical memory, and attachment to the city. This case also highlights the importance of transparency in data journalism. Quantitative stories can appear more definitive than their underlying methodological choices justify, particularly when data provenance and analytical decisions are hidden [17]. In this study, transparency is provided by listing every intersection name in Table 1, specifying how wins, draws, and missing pairs were coded, presenting results from three models rather than one, and displaying both the aggregate ranking and the residual disagreement. These features allow readers to distinguish among the observed fact of the order printed on an intersection sign, the methodological choice of treating the first name as the winner, and the model-dependent inference of the final street ranking. In this sense, the contribution to data journalism lies not simply in using an engaging topic, but in providing a reproducible framework to communicate how quantitative conclusions depend on data construction and method selection.

## Conclusion

This study examined the stability of rankings derived from 59 observed comparisons among 16 Kyoto streets: 51 decisive outcomes and eight draws, with five of the 64 possible pairs missing. The Colley method ranked Kujo-dori first and Nishioji-dori second, whereas both the Keener and Markov chain-based methods ranked Nishioji-dori first. Simple averaging placed Nishioji-dori first, closely followed by Kujo-dori, and averaging ordinal ranks yielded the same top four streets. The mean ranks of Nishioji-dori, Kujo-dori, Higashioji-dori, and Shijo-dori were 4/3, 5/3, 7/2, and 4, respectively.

The HodgeRank-based decomposition assigned 83.91% of the total edge-flow energy to the common gradient component and 16.09% to residual disagreement among methods. Pearson correlations ranged from 0.6558 for Colley–Markov to 0.9058 for Colley–Keener. Thus, the leading group was comparatively stable, but the exact ordering and score spacing remained method-dependent. The analysis underscores the importance of reporting both an overall ranking and the methodological disagreement that the overall ranking conceals. These conclusions are conditional on the selected streets, current intersection names, and the operational rule that name order represents pairwise preference. Within that scope, Nishioji-dori, not Shijo-dori, ranks first in the overall ranking. The novelty of the study lies not in proposing another ranking algorithm, but in integrating three established ranking methods with aggregation and a HodgeRank decomposition to report agreement and localized disagreement together for an incomplete bipartite network. More broadly, the case demonstrates how an accessible local dataset can illuminate general problems of incomplete comparisons, the choice of ranking methods, transparent rank aggregation, and the communication of model-dependent findings to a public audience.

The numerical ranking is not intended to settle cultural preferences among Kyoto’s streets. Sanjo-dori and Gojo-dori occupy the bottom two positions in the present analysis despite their long history, whereas first-ranked Nishioji-dori has the shortest history among the 16 streets analyzed. Many residents may still favor Shijo-dori, which runs through the heart of Kyoto, as the city’s most representative street. These contrasts show why the operational meaning of “stronger” must remain distinct from historical importance, public familiarity, and personal attachment.

Kyoto’s distinctive intersection names and address system have practical value not only for tourists but also for people who live there. The names can evoke the city’s long history and inspire attachment to particular streets and familiar name orders. These practical and cultural meanings help explain why Kyoto’s intersection names attract the interest of a wide audience. By adding a data-science perspective without claiming a single culturally definitive answer, this study offers another way to discuss and appreciate the streets of Kyoto.
